# The Liver under the Spotlight: Bile Acids and Oxysterols as Pivotal Actors Controlling Metabolism

**DOI:** 10.3390/cells10020400

**Published:** 2021-02-16

**Authors:** Charlotte Lefort, Patrice D. Cani

**Affiliations:** Metabolism and Nutrition Research Group, Louvain Drug Research Institute, Walloon Excellence in Life Sciences and BIOtechnology (WELBIO), UCLouvain, Université Catholique de Louvain, Av. E. Mounier, 73 B1.73.11, 1200 Brussels, Belgium; Charlotte.lefort@uclouvain.be

**Keywords:** liver, bile acids, oxysterols, inflammation, gut microbiota, steatosis, cholesterol, lipid metabolism, glucose metabolism

## Abstract

Among the myriad of molecules produced by the liver, both bile acids and their precursors, the oxysterols are becoming pivotal bioactive lipids which have been underestimated for a long time. Their actions are ranging from regulation of energy homeostasis (i.e., glucose and lipid metabolism) to inflammation and immunity, thereby opening the avenue to new treatments to tackle metabolic disorders associated with obesity (e.g., type 2 diabetes and hepatic steatosis) and inflammatory diseases. Here, we review the biosynthesis of these endocrine factors including their interconnection with the gut microbiota and their impact on host homeostasis as well as their attractive potential for the development of therapeutic strategies for metabolic disorders.

## 1. Introduction

The liver, by being the first organ exposed to molecules absorbed from the intestine, plays a vital role in the detoxification of harmful substances (e.g., toxins and xenobiotics) and in the regulation of energy homeostasis [1,2]. This metabolic hub of the body displays an extensive number of signaling pathways mediated by over a dozen of cell types in different proportions with distinct roles [3]. The hepatocytes are the major parenchymal cells of the liver. They are the functional units of this organ and represent 60% of the total liver cells but occupy 80% of the liver volume [4]. These cell types are essential and can manage numerous and various physiological processes (e.g., detoxification, bile acid (BA) synthesis, regulation of glucose and lipid metabolism). Hepatocytes are the biggest cell type of the liver (i.e., 20–40 µm) and have a life span of at least 150 to 200 days in humans and up to 450 days in rodents [4]. Aside from hepatocytes, the liver is also composed of biliary epithelial cells and sinusoidal cells. Specifically, sinusoidal cells, including liver sinusoidal endothelial cells (LSECs), Kupffer cells (KCs) and hepatic stellate cells (HSCs), constitute about 30–40% of total liver cell number and 6.5% of the liver volume [4]. LSECs lie along the sinusoids and have the particularity of possessing pores, also called fenestrae, which mainly filter the molecules of the blood for the hepatocytes [5]. KCs are the resident macrophages of the liver. Under physiological conditions, they act as sentinels and have the capacity, for instance, to clear endotoxins coming from the gut lumen to defend the liver [6]. Finally, HSCs contribute to fibrogenesis and play a major role in the production of the extracellular matrix [3,7]. Altogether, the liver is an intricate organ composed of a complex network of various cell types which need to be properly regulated to maintain an appropriate body homeostasis.

In this review, we will mainly focus on the role played by the hepatocytes on the production of different bioactive lipids such as BAs and oxysterols. We will summarize the major pathways involved in the production of these molecules in both physiological and pathological conditions. We will also report how the oxysterol/BA profiles vary during metabolic and inflammatory disorders and their consequences on the host metabolism. Ultimately, the recent therapeutic strategies designed to tackle metabolic disorders using compounds able to modulate BA metabolism will be delineated.

## 2. Biosynthesis of Oxysterols and BAs

The liver is an organ of utmost importance for the regulation of cholesterol concentration since it is the main tissue involved in both its production and metabolization. At high levels, cholesterol is toxic and can damage the surrounding cells. One efficient way to eliminate this excess from the body is to convert it into its oxidized derivatives, that are oxysterols and BAs which are playing different metabolic roles [8,9]. In fact, the most described function for BAs regards lipid assimilation in the gut. Indeed, after meal ingestion, these molecules are secreted into the duodenum and facilitate the digestion and absorption of dietary fat, steroids and fat-soluble vitamins [8]. However, this role is far from being unique as detailed later in this review.

**Figure 1:**
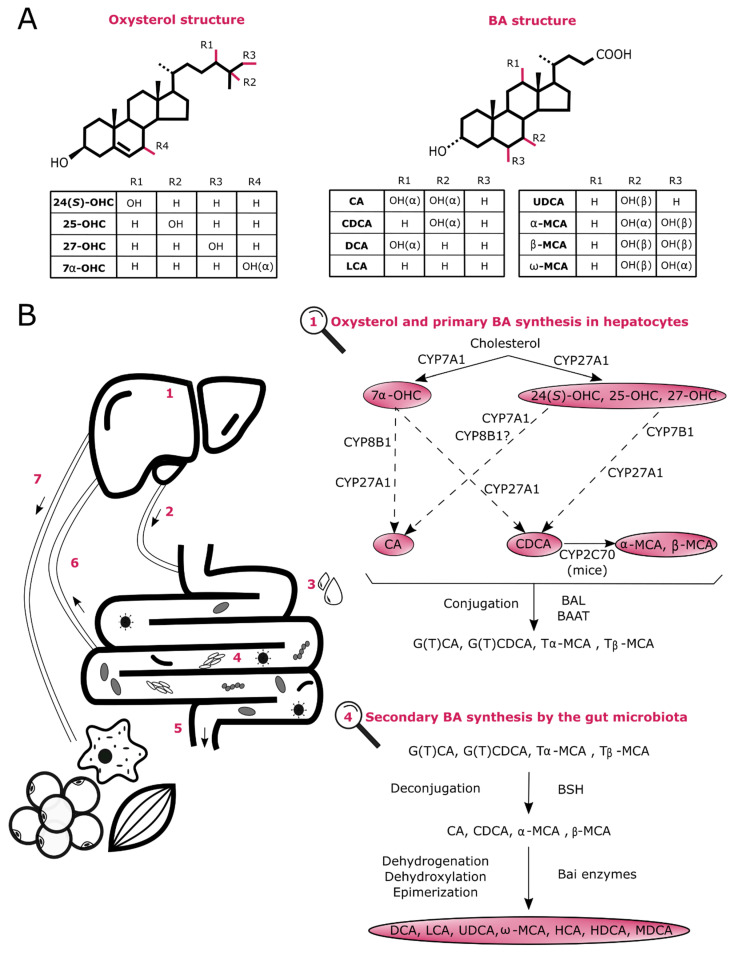
Overview of oxysterol and bile acid (BA) metabolism. (**A**) Structure of the main oxysterols and BAs involved in host homeostasis modulation. (**B**) Biosynthesis and circulation of oxysterols and BAs. (1) Primary BAs are generated from oxysterols through numerous enzymes (e.g., CYP7A1, CYP27A1, CYP7B1 and CYP8B1) in hepatocytes, are then conjugated with T or G by BAL and BAAT and finally stored in the gallbladder. (2) Upon meal ingestion, these are released into the duodenum. (3) BAs can facilitate lipid absorption. (4) Some primary BAs are deconjugated and then converted into secondary BAs by specific intestinal bacteria. (5) While approximately 5% are excreted, (6) about 95% are reabsorbed and travel back to the liver via the portal vein. (7) Finally, a small proportion of BAs reaches other organs (e.g., muscles and adipose tissue) through the systemic circulation. Abbreviations: BA, bile acid; BAAT, bile acid CoA:amino acid *N*-acyltransferase; Bai, bile acid-inducible; BAL, bile acid CoA ligase; BSH, bile salt hydrolase; CA, cholic acid; CDCA, chenodeoxycholic acid; CYP, cytochrome P450 enzyme; DCA, deoxycholic acid; G, glycine-conjugated species; HCA, hyocholic acid; HDCA, hyodeoxycholic acid; LCA, lithocholic acid; MCA, muricholic acid; MDCA, murideoxycholic acid; OHC, hydroxycholesterol; T, taurine-conjugated species; UDCA, ursodeoxycholic acid.

Oxysterols are early oxygenated forms of cholesterol or of its precursors that are generated through enzymatic (e.g., cytochrome (CYP) P450s and cholesterol 25-hydroxylase Ch25OH) and non-enzymatic reactions (e.g., reactive oxygen species (ROS)) [10,11]. Oxysterols resulting from these reactions can be structurally different and divided in several groups including monohydroxycholesterols, dihydroxycholesterols, epoxycholesterols or ketone derivatives (Figure 1A) [12]. Although they were long considered as simple intermediates in the formation of BAs (e.g., 25-hydroxycholesterol (OHC) and 7α-OHC) and steroid hormones (e.g., 22(*R*)-OHC), they also act as signaling mediators. These related activities will be addressed in detail later in this review. Of interest, their synthesis is not restricted to hepatocytes since some have also been detected in immune cells and neurons, among others [12].

Unlike oxysterols, primary BAs are only generated in hepatocytes through an elaborate network involving at least 17 enzymes (Figure 1) [13]. There are two main routes producing primary BAs: the classic and the alternative pathways. In humans, the classic pathway accounts for approximately 90% of total BA production whereas the alternative pathway contributes for the remaining 10% under normal physiological condition. In rodents, however, both cascades participate equally to the synthesis of BAs [14,15].

On the one hand, the classic pathway initiates with the rate-limiting enzyme named cholesterol 7α-hydroxylase (CYP7A1) and aims to produce both cholic acid (CA) and chenodeoxycholic acid (CDCA) [13,16]. The sterol 12α-hydroxylase (CYP8B1) is also a key enzyme of this cascade since it is required to form CA and is, therefore in charge of regulating the CA/CDCA ratio [17]. On the other hand, the alternative pathway regulates oxysterol levels by synthesizing 24(*S*)-OHC, 25-OHC and 27-OHC (also known as 26-OHC) through the transporter steroidogenic acute regulatory protein (StarD1) and the enzyme sterol 27-hydroxylase (CYP27A1) [18]. These bioactive lipids are thereafter metabolized mainly by oxysterol 7α-hydroxylase (CYP7B1) to predominantly generate CDCA. Noteworthy, in rodents, ursodeoxycholic acid (UDCA) is considered as a primary BA and CDCA is mostly converted to α-muricholic acid (MCA) and β-MCA by CYP2C70, a 6β-hydroxylase enzyme recently identified [13,18,19]. Once formed, primary BAs are conjugated with glycine in humans and taurine in rodents by bile acid CoA:amino acid N-acyltransferase (BAAT) and excreted into the biliary canaliculi via the ABC-transporter bile salt export pump (BSEP) to ultimately be stored in the gallbladder [15]. These amphipathic molecules are secreted into the duodenum after a meal but most of the conjugated BAs are then actively reabsorbed in the distal ileum by the apical sodium-dependent bile salt transporter (ASBT), travel back to the liver through the portal blood, are taken up by the Na^+^-dependent taurocholate transporter (NTCP) in hepatocytes to finally be secreted in the gallbladder. This phenomenon is called the enterohepatic circulation. However, a small proportion of BAs escapes this reabsorption and are profoundly affected by the gut microbiota in the ileum and colon. Conjugated BAs are firstly deconjugated by bacterial bile salt hydrolases (BSHs) present in a large variety of bacteria including both Gram-negative (e.g., *Bacteroides*) and Gram-positive genera (e.g., *Clostridium*, *Lactobacillus*, *Listeria* and *Enterococcus*) [20]. Free BAs can either cross the gut barrier through passive diffusion or be further processed by bacterial enzymatic activities (e.g., dehydrogenation and dehydroxylation), increasing the catalogue of BA molecules. Indeed, CA and CDCA are metabolized by the bacterial 7α-dehydroxylase, that removes their 7α-OH group, to form secondary BAs, deoxycholic acid (DCA) and lithocholic acid (LCA), respectively [15]. Additionally, in humans’ gut, CDCA can also be biotransformed into UDCA by 7β-hydroxysteroid dehydrogenase (HSDH) which epimerizes its 7α-OH into a 7β-OH group. Besides, oxo-BAs can also be generated by 3α, 7α and 12α-HSDHs which epimerize the corresponding α-OH group to carbonyl group. These oxo-BAs can be further metabolized into iso-BAs and epi-BAs trough the β-epimerization of the carbonyl group thanks to 3β, 7β and 12β-HSDHs [20]. In mice, murine-specific primary BAs, α-MCA and β-MCA, can be converted into secondary BAs as well. Although both can generate murideoxycholic acid (MDCA), only β-MCA can be metabolized into ω-MCA, hyocholic acid (HCA) and hyodeoxycholic acid (HDCA) [15]. These bacterial metabolites can also be passively absorbed from the gut and can impact the whole organism by acting as signaling molecules (this will be discussed in a subsequent section of this review). In total, ~95% of the total BAs are reabsorbed at some point, whereas ~5% are excreted in the feces [8]. Of interest, in the mouse liver, LCA and DCA can be converted back to CDCA and CA, respectively, through rehydroxylation, by the recently described 7α-hydroxylase (CYP2A12) [19]. Finally, it is worth mentioning that in an adult human liver, around 500 mg of cholesterol is daily catabolized into BAs [13].

## 3. Physiological Roles of BAs on Energy Homeostasis and Inflammation

### 3.1. BA Profile in Inflammatory and Metabolic Diseases

BAs are amphipathic molecules and their hydrophobicity level can be classified according to this order LCA>DCA>CDCA>CA>HDCA>UDCA>β-MCA>α-MCA, free species being more hydrophobic than conjugated BAs (glycine-conjugated> taurine-conjugated) [20,21,22]. One of their primary function regards their ability to digest and absorb lipid-related molecules in the intestine [15]. Nevertheless, in humans, changes in BA profile have also been widely observed in patients suffering from obesity and associated comorbidities (e.g., nonalcoholic fatty liver disease (NAFLD), nonalcoholic steatohepatitis (NASH) and type 2 diabetes (T2DM)). For instance, individuals with metabolic disorders usually have an increase in total BA pool [23]. This has been largely reported in NAFLD/NASH patients in several compartments such as the liver, serum and feces [24,25,26,27]. Besides, the enlargement of the BA pool has recently been associated with the progression of the disease [28,29]. Paradoxically it should be mentioned that an elevated BA pool is not always associated with deleterious health effects given that humans undergoing bariatric surgery are characterized by a rise in BA levels while their metabolic parameters are improving [30]. Moreover, although obesity-related disorders are usually characterized by a chronic low-grade inflammation, individuals with chronic intestinal inflammatory diseases (e.g., Crohn’s disease (CD) and ulcerative colitis (UC)) exhibit another BA profile. Indeed, in inflammatory bowel diseases (IBD), it has been clearly demonstrated that the deconjugation and thus the conversion of primary to secondary BAs were impaired resulting in an increased conjugated primary BA level [31,32,33]. This suggests that the location and severity of inflammation might also influence the BA composition.

**Figure 2:**
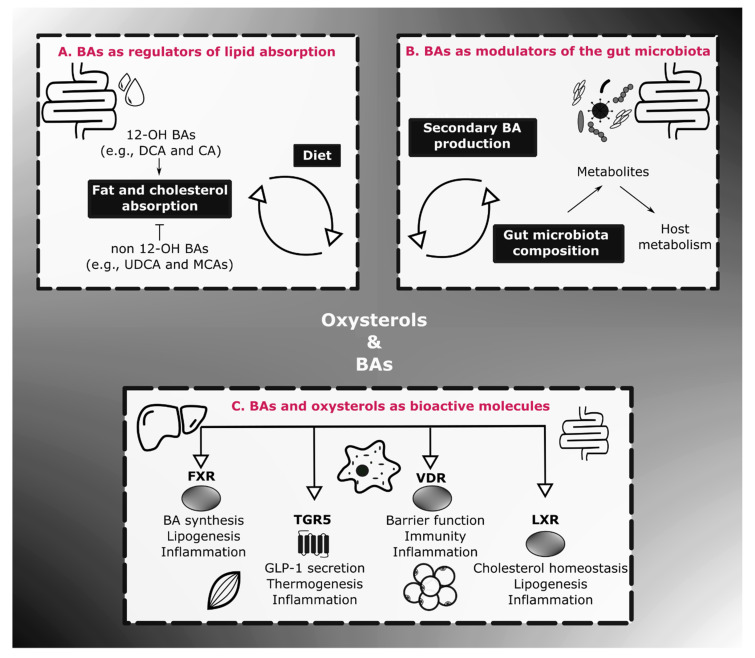
BAs and oxysterols at the nexus of host homeostasis. (**A**) BA profile in the intestine regulates lipid assimilation with 12-OH BAs promoting fat and cholesterol absorption. Conversely, the composition of the diet also influences BA profile since total BAs is increased upon high-fat diet (HFD) exposure. (**B**) A mutual relationship exists between the gut microbiota and BAs. BAs regulate the proliferation, maturation and the composition of the intestinal bacteria while the gut microbiota generates secondary BAs. Displaying a healthy equilibrium is essential since bacterial metabolites including secondary BAs are impacting host metabolism. (**C**) BAs and oxysterols are considered as signaling molecules since they can interact with a panel of receptors distributed in the whole body. The BA receptors FXR, TGR5 and VDR as well as the oxysterol receptor LXR are the most important ones regarding inflammatory and metabolic disorders. Abbreviations: BA, bile acid; CA, cholic acid; DCA, deoxycholic acid; FXR, farnesoid X receptor; GLP-1, glucagon-like peptide 1; LXR, liver X receptor; MCA, muricholic acid; OH, hydroxyl group; TGR5, Takeda G-protein coupled receptor 5; UDCA, ursodeoxycholic acid; VDR, vitamin D receptor.

Coming back to the role of BAs on lipid absorption, it has been established that the composition of BAs released in the intestine influence this function, that can in turn, have an impact on energy homeostasis. Indeed, it has been reported that hydrophobic BAs only produced by the classic pathway and thereby hydroxylated on their C12 (also known as 12-OH BAs), including CA and its microbial-derivative DCA, enhance the absorption of fat and cholesterol in mice by forming efficiently mixed and larger micelles, respectively (Figure 2A) [22,34,35]. This could substantially aggravate metabolic parameters in obesity-related diseases. Conversely, it has recently been demonstrated that UDCA administration in mice, a non 12-OH BA, exerted beneficial metabolic outcomes by increasing the rate of BA enterohepatic circulation and excretion leading to an accelerated BA synthesis and a diminished cholesterol level in the liver [36]. Additionally, by administering BAs from the alternative pathway instead of the classic pathway in mice, it has been demonstrated that an elevated concentration of the hydrophilic non 12-OH BAs, MCAs and UDCA, resulted in a decreased intestinal fat and cholesterol absorption [22]. In line with this, it has been shown that serum 12-OH BAs were augmented in humans with insulin resistance [37]. Moreover, a reduction in non 12-OH BA plasma level was found in unhealthy individuals with high body mass index (BMI) (i.e., ≥25 kg/m^2^) compared to healthy subjects with low or high BMI [38]. Curiously, it should be noted that the ratio 12-OH/non 12-OH rose in the serum of obese who lost weigh in spite of a reduction in their total BA level [39]. Overall, promoting the alternative pathway to manage metabolic disorders seems promising as recently reviewed by Jia and colleagues [40]. However, one should bear in mind that enhancing this alternative pathway may have different consequences on mice compared to humans since CDCA (in humans) and MCA (in mice) exhibit different hydrophobicity level and opposing effects on one BA receptor called farnesoid X receptor (FXR) (this receptor will be extensively discussed later in this review). Further investigations are definitely required to clarify it.

Noteworthy, among all the studies mentioned here above, we noticed some discordances in BA profile associated with one specific disorder. Although this is not clearly addressed in these clinical research, we do believe that these differences may be due to the feeding state of the person (i.e., fasting versus postprandial measurements), the use of drugs influencing the gut microbiota and/or the liver function (e.g., metformin, statins and proton-pump inhibitors), the technique of measurement (e.g., untargeted versus targeted studies), biological sample analyzed (e.g., plasma versus stool), gender and age as well as the severity of the disease.

Collectively, this suggests that BAs might display context-dependent roles and it strengthens the importance of deciphering the exact functions of these bioactive lipids that potentially may be used as future treatments to counteract either inflammatory or metabolic disorders. 

### 3.2. BAs as Endocrine Molecules

Apart from playing a key role in lipid absorption, it is now recognized that BAs act as signaling mediators influencing host homeostasis by interacting with both a G protein coupled receptor named TGR5 (Takeda G-protein coupled receptor 5) and various nuclear receptors such as FXR, vitamin D receptor (VDR), constitutive androstane receptor (CAR) or pregnane X receptor (PXR). While PXR and CAR are mostly associated with drug metabolism and detoxification, it has been proven that FXR, TGR5 and VDR mediate pleiotropic effects in inflammation and energy metabolism (Figure 2C) [14,16].

#### 3.2.1. Farnesoid X Receptor (FXR)

FXR is expressed in various tissues including the liver (i.e., hepatocytes> LSECs, HSCs and KCs), intestine (i.e., enterocytes from ileum > colon), kidneys, adipose tissue, adrenal glands, cardiovascular system and lungs. However, its role has mostly been investigated in the tissues taking part in the BA enterohepatic circulation [16]. FXR is activated by several BAs such as CDCA, DCA, CA and LCA with unconjugated BAs being more potent FXR activators than conjugated BAs [41,42,43,44]. Although this latter fact remains stable across studies, controversial data have been published regarding the ranking of FXR-activating BAs. This difference might vary according to the in vitro/in vivo conditions, organisms, FXR isoforms and sometimes pathological situations. Indeed, several in vitro studies carried out on cell lines from different organisms (e.g., humans and monkeys) have drawn the conclusion that FXR-activating BA rank was the following CDCA > DCA > LCA > CA [41,42,43,44]. In mice, according to an in vivo study investigating the expression of several hepatic and ileal FXR-target genes following the ingestion of specific BA at different doses, CA and DCA were greater FXR activators than CDCA and LCA and differed in a dose-dependent manner [45]. Strikingly, in NASH/NAFLD patients, despite the higher amount of total BAs and consequently FXR agonists, FXR activity is diminished [26,46]. Jiao and colleagues have suggested that DCA might act as FXR antagonist in the presence of CDCA in humans with NASH [26]. It is also worth noting that, in mice, Tα/β-MCAs have been reported to act as FXR antagonists [47].

- FXR and BA Regulation

**Figure 3:**
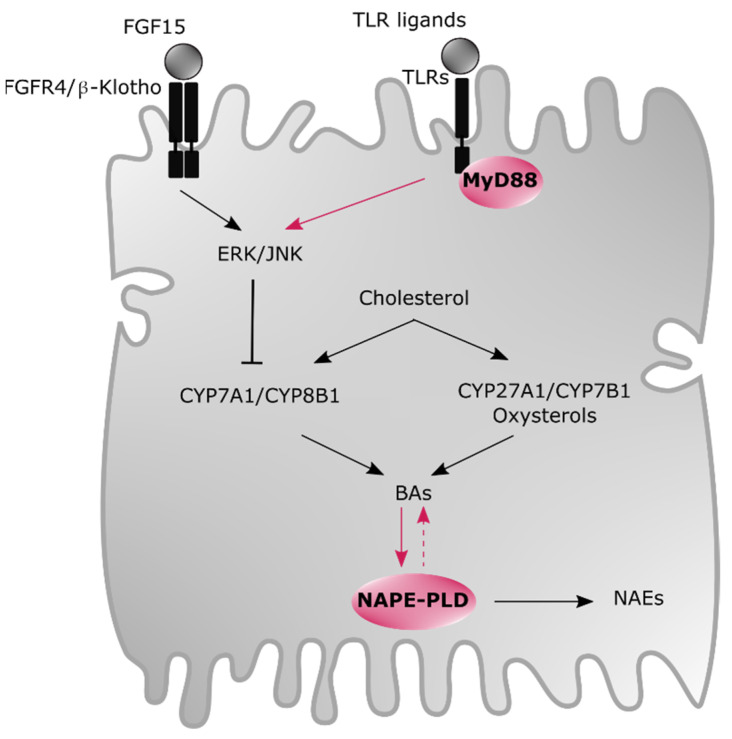
Modulation of oxysterol and BA profiles by the hepatic endocannabinoid and immune system in male mice. BAs are cholesterol-derived bioactive lipids synthesized by two pathways in hepatocytes: the classic pathway (i.e., CYP7A1 and CYP8B1) and the alternative pathway (i.e., CYP27A1 and CYP7B1), this latter being the main route for oxysterol production. BA synthesis is under the regulation of a negative feedback loop. When FGF15, produced by enterocytes and secreted into the portal vein, binds to FGFR4/β-Klotho receptor, it induces the repression of BA production by activating ERK/JNK enzymes. Interestingly, this repression cascade seems also under the control of the immune system involving TLR/MyD88 complex. Finally, a reciprocal regulation might take place between BAs and NAPE-PLD, which is responsible for generating other crucial bioactive lipids named NAEs. Abbreviations: BA, bile acid; CYP27A1, sterol 27-hydroxylase; CYP7A1, cholesterol 7α-hydroxylase; CYP7B1, oxysterol 7α-hydroxylase; CYP8B1, sterol 12α-hydroxylase; ERK, extracellular signal-regulated kinase; FGF15, fibroblast growth factor 15; FGFR4, fibroblast growth factor receptor 4; JNK, c-Jun N-terminal kinase; MyD88, myeloid differentiation primary response gene 88; NAE, *N*-acylethanolamine; NAPE-PLD, *N*-acylphosphatidylethanolamine-selective phospholipase D; TLR, toll-like receptor.

The synthesis of BAs is under the control of a negative feedback loop. When entering in enterocytes, ileal FXR is activated and enhances the expression of both the intestinal bile acid-binding protein (I-BABP) [44], and the organic solute transporter (OST)α and OSTβ that are basolateral BA transporters leading to an efflux of BAs in the portal vein. More importantly, intestinal FXR activation also promotes the secretion of the intestinal fibroblast growth factor (FGF)15 in mice (FGF19 in humans) [14]. Subsequently, this small molecule is conveyed to the portal circulation and reaches the liver to activate the fibroblast growth factor receptor (FGFR)4/β-Klotho receptor that represses CYP7A1 and CYP8B1 through extracellular signal-regulated kinase (ERK) and c-Jun N-terminal kinase (JNK) stimulation leading to the suppression of BA synthesis (Figure 3) [48,49,50]. The inhibition of those two key enzymes is also mediated, to a lesser extent, by the activation of FXR in hepatocytes. This latter will induce the transcription of the nuclear receptor small heterodimer partner (SHP) resulting in the inhibition of CYP7A1 and CYP8B1 to avoid BA accumulation, which can induce liver inflammation and injury [18].

- FXR, Energy Homeostasis and Inflammation

FXR actions have extensively been studied and conflicting results were reported regarding its beneficial effects during pathological conditions. Many *Fxr* knockout animals were generated and several experiments demonstrated that the deletion of *Fxr* was deleterious for the regulation of BA homeostasis as well as lipid and glucose metabolism [51,52,53,54]. First, FXR activation has been indicated to prevent the hepatic accumulation of BAs to toxic levels by inducing BSEP and BAAT in order to enhance BA efflux and conjugation, respectively. In addition to inhibiting BA synthesis, FXR stimulation may also lower the reuptake of plasma BA by downregulating NTCP [55,56,57]. It was also demonstrated that hepatic lipogenesis was decreased upon FXR activation. Indeed, FXR stimulation enhances fatty acid oxidation through peroxisome proliferator-activated receptor (PPAR)α stimulation and reduces de novo lipogenesis by repressing both carbohydrate responsive element binding protein (ChREBP) and indirectly sterol responsive element binding protein 1 (SREBP-1c) [58,59,60,61]. More precisely, hepatic FXR activation leads to the activation of SHP which in turn inhibits liver X receptor (LXR) resulting in the repression of SREBP-1c [62]. Regarding glucose homeostasis, the role of FXR is less clear. One study showed that FXR stimulation inhibited gluconeogenesis by repressing two rate-limiting step enzymes (i.e., phosphoenolpyruvate carboxykinase (*Pck1*) and glucose-6-phosphatase (*G6pc*)) in vitro whereas another study demonstrated the opposite [63,64]. In spite of this dissimilarity, most of the experiments performed on mice indicated that FXR activation lowers blood glucose level and enhances insulin sensitivity [53,54,65]. Finally, anti-inflammatory properties have also been described upon FXR activation in the liver. Although, the decrease in proinflammatory cytokines is likely due to the transrepression of nuclear factor-kappa B (NF-κB) [66,67,68], other mechanisms may also occur. For instance, FXR anti-inflammatory effects could also be linked to a reduced hepatic lipid accumulation [61]. Indeed, an elevation of hepatic lipids has been reported to induce ROS formation and to activate NF-κB-related pathways, worsening liver inflammation [69,70]. Further studies are thereby required to provide a better understanding of the mechanisms underlying this FXR function.

After having introduced the positive effects of FXR activation, it should be noted that its inactivation can also be beneficial in metabolic diseases [71,72]. For instance, Prawitt and colleagues demonstrated, in mice, that the deletion of *Fxr* conferred a protection against insulin resistance as well as obesity induced either genetically or by the diet [71]. Additionally, another study indicated that upon FXR agonist administration, mice fed with a high-fat diet (HFD) gained more weight [73]. In view of all these inconsistencies, researchers went further and succeeded in generating organ-specific FXR knockout mouse models as well as specific (ant)agonists in an effort to assess the tissue-dependent FXR functions. Nonetheless, by genetically disrupting, inhibiting (i.e., glycine-β-MCA) or enhancing (i.e., fexaramine) only intestinal FXR, here again, paradoxical effects were reported [74,75,76,77]. Interestingly, Schmitt and coworkers suggested that hepatic FXR activation would rather be protective since its specific-liver deletion led to an increase in hepatic lipid accumulation under cholesterol diet [78]. Additional studies are clearly warranted to shed light on the beneficial versus deleterious effects of FXR activation in various tissues and different pathological conditions.

#### 3.2.2. Takeda G-Protein Coupled Receptor 5 (TGR5)

TGR5 is widely expressed in metabolic relevant tissues such as brown adipose tissue (i.e., adipocytes), pancreas (i.e., β-cells), intestine (i.e., L-cells and monocytes), muscles (i.e., skeletal and smooth), gallbladder and the liver (i.e., KCs and cholangiocytes) [79,80]. Its strongest endogenous agonist includes LCA and, to a lesser extent, (un)conjugated DCA, CDCA, UDCA and CA [81,82]. Interestingly, TGR5 activation promotes health benefits through different mechanisms of action. First, it impacts mitochondrial energy homeostasis by increasing thermogenesis in muscles and adipose tissues [83,84]. Then, it promotes the release of the incretin glucagon-like peptide 1 (GLP-1) in enteroendocrine cells of the gut enhancing insulin secretion [85,86]. Finally, it contributes to the reduction of inflammation in both the liver by inhibiting the nuclear translocation of NF-kB in KCs [87,88] and in the intestine in IBD-related context [80,89].

#### 3.2.3. Vitamin D Receptor (VDR)

VDR is expressed in various cell types of the immune system (e.g., lymphocytes, neutrophils, macrophages and dendritic cells) and in organs of metabolic relevance including the liver, adipose tissue and intestine [90,91]. This receptor was primary known to be stimulated by the active form of vitamin D (i.e., 1,25-dihydroxyvitamin D3 (1,25(OH)2D3)), and later by LCA [91,92,93]. Nowadays, it is established that VDR modulates immunity, gut barrier integrity and inflammation [90,91,93]. For instance, VDR activation by LCA exerts anti-inflammatory action in colonic cancer cells by repressing NF-kB signaling [94]. This is consistent with the fact that VDR activation by 1,25(OH)2D3 also mediates anti-inflammatory properties [95,96]. More recently, it has been reported that specific LCA-derived molecules (i.e., 3-oxoLCA and isoalloLCA) influence intestinal host immunity through VDR receptor [97,98]. Finally, in 2020, Chatterjee and coworkers explored the impact of the deletion of *Vdr* in intestinal epithelial cells and in myeloid cells, on both the gut microbiota and their associated metabolites. They discovered that these deletions deeply impacted 84 among the 765 metabolites analyzed and sometimes in a gender-dependent manner. For instance, the secondary BAs, LCA and DCA, were found increased in the feces of females deleted for *Vdr* and not in males suggesting that sex hormones might influence BA profile. BA metabolism was further examined and both intestinal and hepatic FXR protein expression were elevated following *Vdr* disruption. This increase was even higher when exposing the mice to HFD [99]. Altogether, these studies highlight the relevance of better understanding the function of VDR especially regarding metabolic and inflammatory diseases.

### 3.3. BAs, Gut Microbes and Energy Homeostasis

In addition to acting as signaling factors, BAs can also modulate host homeostasis directly and indirectly via the gut microbiota (Figure 2B). Indeed, as described earlier in this review, several gut microbes are able to directly convert primary BAs into secondary BAs through the enzymes BSH and 7α-dehydroxylase, among others [20,100]. Interestingly, one resulting metabolites, LCA holds a particular interest in metabolic and inflammatory disorders since it is the more potent agonist ligand of three BA receptors (i.e., TGR5, PXR and VDR) influencing positively the host metabolism [82,92,101,102,103]. It is thereby not surprising that the reduction of secondary BAs has been associated with health disorders such as chronic intestinal inflammatory diseases (i.e., CD and UC) [31]. This emphasizes another key role of the gut microbiota and eventually the importance of having an appropriate gut ecosystem in pathological situations (for review [83,104]). With this in mind, Allegretti and colleagues conducted a pilot study in which overweight individuals (BMI > 25kg/m^2^) received fecal microbiota transplantation (FMT) from a single lean donor (BMI = 17.5 kg/m^2^) by oral capsules during 12 weeks. Although their BMI did not change, they exhibited a “normalized” BA profile strengthening the impact of the gut microbiota on the modulation of BA profile [105]. Finally, recent studies shed light on the fact that some of these microorganisms also participate to the metabolization of cholesterol, which is the precursor of BAs, through specific enzymes [106,107]. Indeed, Kenny and colleagues identified a group of bacterial cholesterol dehydrogenases encoded by *ismA* genes that convert cholesterol to coprostanol, the latter being mostly excreted in feces [107].

On the other hand, the indirect way includes the modulation of the gut microbiota by BAs. Indeed, these bioactive molecules can alter the maturation, composition and proliferation of these microorganisms, by notably exhibiting antimicrobial properties [108,109,110]. Therefore, since the gut microbiota is a central actor driving host homeostasis by producing a multitude of metabolites [83], exhibiting a proper BA profile seems crucial to avoid health complications.

Altogether, the existence of this mutual crosstalk is captivating and should receive further attention when exploring host physiology.

## 4. Oxysterols in Energy Homeostasis and Inflammation

### 4.1. Oxysterol Profile in Inflammatory and Metabolic Diseases

Level of oxysterols is altered under pathophysiological conditions such as inflammatory diseases (e.g., IBD), obesity-related disorders (e.g., NAFLD and T2DM) and some cancers [111,112,113,114,115,116,117,118]. Oxysterol measurement from colon biopsies of IBD patients showed that 25-OHC level was higher while 4β-OHC level was lower compared to healthy individuals [118]. Although this field of research is still in its infancy, more data are available regarding metabolic disorders. For instance, a reduction of serum 4β-OHC level has been associated with obesity in humans [111]. Accordingly, this diminution has also been noted in the liver and adipose tissue of both genetically and diet-induced obese mice [112]. Conversely, 4β-OHC as well as 25-OHC and 27-OHC were increased in the blood of NAFLD patients compared to control individuals [116]. Consistent with this finding, hepatic disorders such as cirrhosis were also observed in humans with *Cyp7b1* mutation and were associated with a plasma accumulation of 24(*S*)-OHC, 25-OHC and 27-OHC [119,120,121]. Noteworthy, this oxysterol profile might evolve with the disease progression of NAFLD to NASH or might be different according to the samples harvested for oxysterol measurement. Indeed, in a recent study, Raselli and colleagues measured in liver samples, an increased level of 24(*S*)-OHC and 7-OHC derivatives in NASH patients compared to controls [122]. In line with this, this profile was also identified in the liver of murine model of NASH [122]. Regarding patients with diabetes or hyperlipidemia, a higher 25-OHC, 27-OHC and 7-KC plasma levels were reported compared to healthy controls [114]. Finally, although an excess of some oxysterols is correlated with metabolic disorders, it is worth mentioning that the absence of oxysterols from the alternative pathway also results in devastating health condition. This is the case for humans harboring *Cyp27a1* mutation which have an elevated cholesterol level and suffer from cerebrotendineous xanthomatosis [123,124].

### 4.2. Oxysterols as Endocrine Molecules

Oxysterols have been described to exert various effects on host homeostasis through numerous molecular targets including LXR, insulin-induced gene (INSIG) proteins, the Epstein–Barr virus-induced gene 2 (EBI2, also known as GPR183), Smoothened (SMO), the retinoid-related orphan receptor (ROR) and the estrogen receptor (ER)α [9,10,125,126]. Although the modulation of immunity by oxysterols via ROR, ER or GPR183 is an interesting topic [10,12], in this review, we will focus on their roles in energy homeostasis and inflammation principally via LXR (Figure 2C).

#### Liver X Receptor (LXR)

LXR family is involved in the regulation of cholesterol homeostasis, BA synthesis, glucose and lipid metabolism as well as in inflammation [127]. This family includes two isotypes, LXRα and LXRβ, and commonly forms a heterodimer with the retinoid X receptor (RXR)α. LXRα is expressed in metabolically active tissues (e.g., liver, adipose tissue and intestine) whereas LXRβ is ubiquitously expressed [128]. Noteworthy, it has been discovered that their transcriptional activity was regulated by desmosterol, a precursor of cholesterol, and several oxysterols such as 24(*S*)-OHC, 25-OHC, 27-OHC and 24(*S*), 25-epoxycholesterol [125,127].

- LXR and Cholesterol Homeostasis

It has been largely recognized that oxysterols mediate the elimination of cholesterol excess by activating LXR and by inhibiting SREBP. In response to a low cellular level of cholesterol, SREBP is activated and is responsible of the synthesis and uptake of cholesterol by inducing the transcription of 3-hydroxy-3-methylglutaryl coenzyme A reductase (*Hmgcr)* and low-density lipoprotein receptor (*Ldlr*), among others [125,129]. Conversely, when the concentration of cellular cholesterol is high, SREBP is retained in the endoplasmic reticulum (ER) due to the binding of cholesterol/desmosterol and some oxysterols (e.g., 24(*S*)-OHC, 25-OHC and 27-OHC) on SREBP-cleavage activating protein (SCAP) and INSIG respectively. This action suppresses the synthesis and the uptake of cholesterol. At the same time, oxysterols and desmosterol interact with LXR resulting in the enhancement of the excretion of cholesterol by inducing ATP-binding cassette(ABC) subfamily A member(ABCA)1 and ABC subfamily G member(ABCG)1 and in the inhibition of its uptake by stimulating the inducible degrader of the low-density lipoprotein receptor (IDOL) [125]. Moreover, LXR further mediates the elimination of cholesterol excess by inducing the transcription of *Abcg5/8* and *Cyp7a1* in order to increase its efflux and conversion to BAs, respectively [130,131].

- LXR, Glucose and Lipid Metabolism

Interestingly, LXR activation by some of its oxysterol agonists (e.g., 22(*R*)-OHC and 24(*S*),25-epoxycholesterol) enhances de novo lipogenesis by inducing the expression of notably *Srebp1c*, fatty acid synthase (*Fasn*) and stearoyl-CoA desaturase *(Scd1*) [128,132,133,134]. In addition, LXR stimulation leads to the inhibition of gluconeogenesis by decreasing the expression of *Pck1* and *G6pc* [135,136]. Of interest, a similar reduction has been indicated in murine primary hepatocytes treated with 7α-OHC, but this time, in a ROR-dependent manner [137].

The involvement of LXR in glucose and lipid metabolism has been further confirmed by using a non-endogenous oxysterol (i.e., 22(*S*)-OHC) which behaves as a LXR antagonist [126]. Indeed, incubation of 22(*S*)-OHC with human skeletal muscle cells from lean, obese and type 2 diabetic individuals enhanced glucose uptake and decreased lipogenesis as reflected by the reduced gene expression of *Fasn* and *Scd1* in all groups [138]. Noteworthy, the reduction of lipid accumulation and *Fasn* mRNA expression have also been observed in murine adipocytes treated with 27-OHC [139].

- LXR and Inflammation

One last LXR function discussed in this review regards its impact on inflammation. LXR stimulation leads to the reduction of the inflammatory response and several mechanisms have been proposed over the past few years. As such it has been suggested that LXR may suppress inflammation through the transrepression of proinflammatory gene promoters, the promotion of cholesterol efflux, the alteration of lipid profile resulting in an increase in anti-inflammatory lipid level and through the modification of immune cell phenotype [140]. However, although the influence of cholesterol efflux in decreasing inflammation seems to be consistent across studies, it is worth noting that the transrepression activity has been challenged and that Thomas and colleagues recently discovered a *cis*-repressive activity linked to LXR [141,142]. More studies are definitely required to decipher the exact molecular action of LXR in suppressing inflammation.

In line with this, Jakobsson and coworkers demonstrated that mice deleted for *Lxr* were more prone to develop colitis compared to controls and that this intestinal inflammation was diminished upon LXR agonist administration. In addition, they showed that inflamed colon of IBD subjects had lower *Lxr* mRNA expression level compared to non-inflamed colons [143]. Finding the right oxysterol(s) leading to this anti-inflammatory effect may open new therapeutic strategy to treat chronic intestinal inflammatory diseases. Nevertheless, one should keep in mind that activating LXR might also lead to increased lipogenesis and different LXR antagonists are being investigated to tackle NAFLD (e.g., 25-OHC-3S) [144,145,146]. Therefore, intending to design an intestinal-specific LXR agonist might be interesting in this context.

Finally, it should be mentioned that 25-OHC, which can bind to a wide spectrum of receptors (e.g., LXRα, LXRβ, GPR183, RORα and RORβ) [147,148,149], is the most studied oxysterol regarding inflammation. However, its function is still debated since it exhibits both pro- and anti-inflammatory properties [150,151]. This complexity is further underscored by a recent study of Guillemot-Legris and colleagues which indicated that 25-OHC administration had no inflammatory effect in the colon of a colitis mouse model. Still, they surprisingly found that 4β-OHC administration worsened the intestinal inflammation of this mouse model supporting a potential new function for this oxysterol [118]. Besides this study, we observed that in two specific mouse models of genetically induced hepatic inflammation, both 25-OHC and 4β-OHC, were either strongly affected or unaltered despite a similar liver inflammatory tone [152,153]. Altogether, these data support that further experiments are needed to clarify the exact roles of these two oxysterols in inflammation and eventually the molecular targets of 4β-OHC.

To conclude, despite their structural similarities, oxysterols exhibit a broad range of physiological effects and sometimes show opposite actions. It should be emphasized that it is quite difficult to assign a clear function to a specific oxysterol since this system is highly complex. Indeed, one oxysterol can target several receptors and these receptors are not specific to one oxysterol. Moreover, a single enzyme can be involved in the formation of several oxysterols (e.g., CYP27A1 and CYP7B1) and a specific oxysterol can either be generated by different pathways (e.g., 25-OHC) or metabolized through various enzymes (e.g., 7α-OHC and 27-OHC) [9]. Nonetheless, given their involvement in key signaling pathways associated to inflammatory and metabolic disorders, research on these bioactive lipids should definitely be pushed forward.

## 5. Newly Identified Modulators of BAs and Oxysterols

Although the synthesis and the regulation of both BAs and oxysterols are becoming well described in the literature, we have discovered that disrupting specific genes involved in innate immunity or belonging to the endocannabinoid system, within hepatocytes, was strongly linked with a modulation of both the synthesis and the degradation of BAs and oxysterols (Figure 3) [152,153].

Myeloid differentiation primary response gene 88 (MyD88), a key player of the immune system, has long been considered as only controlling inflammatory signaling cascades. However, its physiological role has evidently been undervalued. More precisely, we previously demonstrated that MyD88 was also able to modulate energy, glucose and lipid metabolism [154]. Recently, we discovered that mice harboring hepatocyte-specific deletion of MyD88 (*Myd88*^∆*Hep*^) were predisposed to liver fat accumulation, glucose intolerance and inflammation [155]. By further exploring the molecular mechanisms underlying this phenotype, we performed lipidomic analysis and found that *Myd88*^∆*Hep*^ mice had an altered BA and oxysterol metabolism [153]. We showed that the absence of MyD88 in hepatocytes impacted the negative feedback loop suppressing BA synthesis likely by a mechanism involving ERK activity. Finally, we observed that the predisposition of these mice to hepatic inflammation was linked to the accumulation of 25-OHC and to a lower extent to 4β-OHC [153]. Altogether, these data revealed an unexpected crosstalk between the innate immune system and liver lipid metabolism and highlighted a new role for hepatocyte MyD88 as a regulator of BA synthesis.

The endocannabinoid system participates to the regulation of several crucial functions in host health such as food intake, energy balance and inflammation [156,157]. Accordingly, alteration of this complex system has been associated with diverse metabolic disorders. Remarkably, among the bioactive lipids involved in this system, modifications of *N*-acylethanolamine (NAE) levels have been reported. The role of these bioactive lipids are emerging and they are mainly synthesized by *N*-acylphosphatidylethanolamine-selective phospholipase D (NAPE-PLD) [156].

We have previously proven that deleting *Napepld* in either the intestine or the adipose tissue was correlated with a higher susceptibility to obesity, diabetes and inflammation [158,159]. In addition, by generating a new mouse model of inducible *Napepld* hepatocyte-specific deletion (*Napepld*^∆Hep^), we found that the absence of NAPE-PLD, specifically in hepatocytes, induced an increased fat mass gain and hepatic steatosis in mice and that *Napepld*^∆Hep^ mice were more sensitive to liver inflammation compared to controls [152]. By seeking for the molecular mechanisms, as expected we found that the majority of the endocannabinoids were affected by the deletion. More surprisingly, by using a lipidomic approach we discovered that *Napepld*^∆Hep^ mice displayed a distinct profile of both oxysterols and BAs [152]. We thereby identified a novel role for hepatocyte NAPE-PLD which goes beyond the mere synthesis of NAEs. Interestingly, it has been recently demonstrated that BAs also regulate NAPE-PLD activity suggesting the existence of a potential mutual crosstalk between the endocannabinoid system and BA metabolism. As such, DCA (K_D_ ~43 µM) and CDCA (K_D_ ~25 µM) have been described to stabilize and drive NAPE-PLD catalytic activity whereas LCA (K_D_ ~20 µM), which showed the highest affinity for the enzyme, has been indicated to inhibit its activation [160]. In line with this, in vitro LCA inhibits the enzyme at a low concentration (~68 µM) whereas the BA concentration required to activate NAPE-PLD and to induce a half-maximal response varies from ~2 to 4 mM [160]. Although the physiological meaning of this study remains to be assessed in vivo, these results seem promising since BA concentration spans from ~2 to 10 mM in the ileum after meal ingestion and at lower range (µM) in the blood and the liver [26,161,162]. More recently, tauroursodeoxycholic acid (TUDCA), taurohyodeoxycholic acid (THDCA) as well as α/β-MCAs and their taurine-conjugated forms have also been identified as NAPE-PLD inhibitors, although slightly less potent than LCA [163]. These studies paved the way to the design of specific NAPE-PLD modulators [164].

Even though the exact mechanisms explaining the interconnections observed between either the endocannabinoid or immune system and BAs/oxysterols are still under investigations, these data strongly suggest that any putative dietary intervention or drug treatment targeting the immunity, or the endocannabinoid system might influence BA and/or oxysterol profile, and thus host homeostasis.

## 6. Therapeutic Strategies

Metabolic and inflammatory disorders are still rising among the worldwide population [165,166]. Despite huge efforts to slow their progression, the efficacy of the current treatments is still limited emphasizing the need to find new therapeutic approaches [165,167]. As BAs are bioactive lipids displaying pleiotropic actions regarding energy homeostasis and inflammation, several strategies have been developed based on their metabolic functions. Although this is not the purpose of this review, we have briefly mentioned the current targets and strategies.

Due to its pivotal function on host homeostasis, FXR has become an attractive therapeutic target to treat metabolic disorders and many FXR agonists have been designed [168]. Among those, obeticholic acid (OCA), a semi-synthetic BA analogue based on CDCA structure which has already been approved to treat primary biliary cholangitis in humans, showed all the beneficial health effects of FXR activation when administered in animals [66,169,170,171] and is currently a good candidate for the treatment of NASH and T2DM [16]. From a clinical point of view, administration of OCA in a phase II trial in NAFLD and T2DM patients resulted in an amelioration of insulin sensitivity and a decrease in liver proinflammatory markers [172]. Moreover, in a phase III clinical trial for treating NASH, the administration of 25 mg of OCA on a daily basis during 18 months improved fibrosis in NASH patients [173]. Noteworthy, some safety concerns have been raised regarding this compound such as an increased level of serum LDL cholesterol, decreased level of serum HDL cholesterol, gastrointestinal issues and pruritus. These side effects have delayed its approval on the market and other novel candidates are currently under clinical investigations [168,174].

Aside from FXR, TGR5 is also a potential interesting target. Indeed, many rodent studies showed that TGR5 activation reduces inflammatory responses and promotes thermogenesis and insulin sensitivity. Hence, this receptor has drawn considerable attention from a therapeutic view [16,79]. Over the past years many selective and dual agonists (i.e., TGR5/FXR) have been developed and tested [86,175,176,177,178,179,180]. Despite extensive efforts, the majority of these compounds triggered unwanted side effects mainly because TGR5 is widely expressed in the body and has different physiological actions [79]. For instance, TGR5 over-activation caused pruritus, nausea and gallbladder filling in rodent studies [181,182,183]. Moreover these adverse effects might be due to the fact that these agonists potentially display off-target effects by stimulating other receptors including LXR and PXR [147,184,185] and exhibit detergent-like properties [186]. For all of these reasons, the focus of the current studies has shifted toward the identification of non-steroidal intestinal-selective TGR5 agonists to tackle T2DM even though avoiding the systemic exposure restricts the beneficial effects of TGR5 regarding energy expenditure and inflammation [187,188,189]. Finally, it should be pinpointed that both clinical studies and evidence of beneficial effects of TGR5 activation in humans are still scarce and required further investigation [79].

In parallel to the design and exploration of receptor activity modulators, FGF19 analogs are currently studied. Based on its ability to both suppress BA synthesis and modulate energy homeostasis, FGF19 has recently emerged as an interesting candidate to treat metabolic disorders [190,191,192]. However, FGF19 has also been associated with an increased risk of developing cancer [193,194]. Consequently, an engineered FGF19 analog, Aldafermin, which does not promote tumorigenesis, has been developed and tested in NASH clinical trial. This molecule shows great potential since in a phase II study comprising 78 patients with NASH, it has been reported that patients receiving daily 1 mg of Aldafermin for 24 weeks had a reduced hepatic lipid content.

Finally, the use of BA sequestrant (e.g., colesevelam and colestimide) or ASBT inhibitors, to limit BA absorption and FXR activation, have been investigated but this strategy may be a doubled-edged sword. It is recognized that BA sequestrant improves glucose metabolism and lowers cholesterol level by enhancing its conversion to BAs in the liver but it might also promote an elevation of plasma triglycerides and hepatic steatosis [23,195,196]. Of interest, although displaying similar effects regarding cholesterol metabolism, ASBT inhibitors only partially restrict the absorption of BAs since free BAs are still able to cross the gut barrier through passive diffusion allowing, at a lesser extent, their endocrine actions [197]. Accordingly, its administration in animal models fed with HFD resulted in an improvement of metabolic parameters regarding hepatic steatosis and insulin sensitivity [197,198]. In the future, research should assess its putative therapeutic efficacy in humans with metabolic disorders such as NAFLD or T2DM [199,200].

## 7. Conclusions and Perspectives

Overall, the liver is a vital and metabolically complex organ that contributes to the synthesis of a constellation of molecules that take part in the regulation of host homeostasis. As discussed in this review, among the plethora of bioactive compounds endogenously produced, BAs and oxysterols are very intricate bioactive lipids acting through various receptors present in numerous tissues. Their synthesis and degradation are finely tuned and controlled by different mechanisms among which some are still to be discovered. Nevertheless, BAs and to a lesser extent, oxysterols, are currently under investigation in preclinical and clinical studies and both are appearing as emerging therapeutic targets to tackle inflammatory and metabolic disorders.

We do believe that future therapies will successfully reach the market, but we also want to highlight that cautious interpretations are warranted regarding the comparisons between data obtained in mice versus humans. This is obviously the case for all the research performed in medicine, but the very specific profile of BAs observed in mice versus humans and eventually the putative changes in receptor affinity and expression may be additional interfering factors. Finally, besides the considerations of the living organism, gender differences are also very critical confounders when investigating metabolic diseases and BA metabolism.

Ultimately, all data deeply demonstrate the existence of multiple interconnections and redundant pathways between cholesterol metabolism, gut microbiota and host homeostasis. Hence, underlining the importance of considering all these systems when working in the field of metabolism and nutrition.
